# SENIORLAB: a prospective observational study investigating laboratory parameters and their reference intervals in the elderly

**DOI:** 10.1097/MD.0000000000005726

**Published:** 2017-01-10

**Authors:** Martin Risch, Urs Nydegger, Lorenz Risch

**Affiliations:** a Kantonsspital Graubünden, Chur; b Labormedizinisches Zentrum Dr. Risch, Liebefeld bei Bern, Bern, Switzerland; c Private University Triesen, Liechtenstein; d University of Bern, University Institute of Clinical Chemistry, Bern, Switzerland.

**Keywords:** laboratory parameters, morbidity, mortality, observational study, quality of life, reference intervals, senior, study protocol

## Abstract

**Background:**

In clinical practice, laboratory results are often important for making diagnostic, therapeutic, and prognostic decisions. Interpreting individual results relies on accurate reference intervals and decision limits. Despite the considerable amount of resources in clinical medicine spent on elderly patients, accurate reference intervals for the elderly are rarely available. The SENIORLAB study set out to determine reference intervals in the elderly by investigating a large variety of laboratory parameters in clinical chemistry, hematology, and immunology.

**Methods/design:**

The SENIORLAB study is an observational, prospective cohort study. Subjectively healthy residents of Switzerland aged 60 years and older were included for baseline examination (n = 1467), where anthropometric measurements were taken, medical history was reviewed, and a fasting blood sample was drawn under optimal preanalytical conditions. More than 110 laboratory parameters were measured, and a biobank was set up. The study participants are followed up every 3 to 5 years for quality of life, morbidity, and mortality. The primary aim is to evaluate different laboratory parameters at age-related reference intervals. The secondary aims of this study include the following: identify associations between different parameters, identify diagnostic characteristics to diagnose different circumstances, identify the prevalence of occult disease in subjectively healthy individuals, and identify the prognostic factors for the investigated outcomes, including mortality.

**Discussion:**

To obtain better grounds to justify clinical decisions, specific reference intervals for laboratory parameters of the elderly are needed. Reference intervals are obtained from healthy individuals. A major obstacle when obtaining reference intervals in the elderly is the definition of health in seniors because individuals without any medical condition and any medication are rare in older adulthood. Reference intervals obtained from such individuals cannot be considered representative for seniors in a status of age-specific normal health. In addition to the established methods for determining reference intervals, this longitudinal study utilizes a unique approach, in that survival and long-term well-being are taken as indicators of health in seniors. This approach is expected to provide robust and representative reference intervals that are obtained from an adequate reference population and not a collective of highly selected individuals.

**Trial registration:**

The present study was registered under International Standard Randomized Controlled Trial Number registry: ISRCTN53778569.

## 1 Background

In clinical medicine, decisions are often made based on patient history and examination results. Based on such types of information, prognosis and disease course can be estimated, and treatment decisions can be made.^[1]^ Within this context, laboratory medicine frequently provides important supportive information, on which decisions are based. In approximately 50% to 79% of hospitalized patients or outpatient cases from general internal medicine providers, laboratory measurements play a decisive role when planning the treatment course for the patient.^[2–4]^

Laboratory test results are interpreted in the context of clinical information by comparison with reference intervals and longitudinal interpretation of sequential results.^[5–7]^ These reference intervals are most often obtained from investigations in healthy adult persons aged <60 years.^[8,9]^ Although elderly persons sometimes are regarded as elderly versions of adults, from a physiopathological viewpoint, seniors—analogously to children—have their distinct own biology. Despite this, reference intervals obtained from young and healthy persons are often used in elderly patients.^[10–12]^

The importance of building reliable and robust reference intervals in laboratory parameters has recently gained increasing attention.^[13]^ In 2005, the International Federation of Clinical Chemistry and Laboratory Medicine founded a working group on reference ranges, and its publications have laid a foundation for the investigation of reference intervals.^[13–15]^ Although the majority of health care resources are spent on the elderly, reference ranges for the elderly are scarce. Thus far, with the exception of the very recently published data obtained within the framework of the Canadian Health Measures Survey,^[16–18]^ few studies have aimed to define normal ranges for the most common analyses, which included <40 parameters in each study.^[19–26]^ Some of this scientific work has been published >20 years ago and has been conducted in relatively small cohorts. As a major drawback of these studies, the employed laboratory methods cannot be traced to reference methods or are not in use anymore. Furthermore, all of these investigations are cross-sectional in nature. Accordingly, the usefulness of the reference intervals of the reported parameters can be considered to be limited.

Wrong interpretation of laboratory test results due to the use of unsuitable reference ranges leads to false-negative and false-positive results. False results are a major source of harm in a patient. Time-consuming and worrying test procedures are too often performed in medicine to substantiate (false) positive findings. These procedures are not only burdensome for the patient and his or her family caregivers but also a major cause of unnecessarily expenses. At the end of life, the aim is to limit medical procedures to the minimum. Unnecessary procedures in this phase of life can be considered unethical because they put an unnecessary burden on the shoulders of a patient whose life should be made as comfortable as possible. As long as clinical medicine uses inappropriate reference intervals for the elderly, unnecessary procedures may be commonly ordered. More appropriate reference intervals are expected to reduce the frequency of potentially humiliating medical procedures during the last phase of life.^[1]^

To obtain better grounds on which to base clinical decisions, specific reference intervals for frequently used laboratory parameters of the elderly are urgently needed.^[10–12]^ The present study protocol presents the SENIORLAB study as an investigation with an extensive examination of study participants at baseline and, as a further unique feature of this study, a longitudinal follow-up.

The study setting allows for work on several aims and investigation of a multitude of research questions. The primary aim is to establish robust reference intervals for >110 different parameters in subjectively healthy elderly persons who demonstrated survival at the follow-up examinations. This additional methodological strength of the SENIORLAB study is unprecedented and has the potential of modifying the theory of constructing reference intervals within the senior citizen community. The secondary aims of the SENIORLAB study are as follows: to test the diagnostic characteristics of certain laboratory parameters (e.g., red blood cell characteristics) and to characterize biochemically defined disorders such as vitamin B_12_ deficiency, which can also occur in a subjectively healthy elderly cohort (this approach may help identify novel markers for more efficient identification of common disorders among the elderly population); to investigate associations of different biomarkers with other biomarkers (this approach helps to better understand the pathophysiology and behavior of the different laboratory parameters in seniors and may ultimately lead to a better interpretation of laboratory results, preventing false-negative and false-positive diagnosis in elderly patients with the subsequent consequences); to investigate the prevalence of occult and early disease in subjectively healthy elderly persons (e.g., type 2 diabetes mellitus, thyroid disease, vitamin deficiency, renal disease) (some disorders are exclusively diagnosed by laboratory parameters; this approach helps estimate the magnitude of relevant underdiagnosed diseases in the elderly and may help shape policies regarding better and earlier detection for earlier intervention); and to investigate risk factors for the loss of subjective well-being, mortality, hospitalization, and impaired autonomy among the elderly. Identifying these factors may help identify elderly individuals who may appear healthy but require closer medical attention and may benefit from medical interventions.

## 2 Methods/design

### 2.1 Study design

Reporting the design of this study strictly follows the STROBE statement, except for the guidelines in results and discussion sections because this report is a study protocol.^[27]^ The research project is designed as a prospective, single-center, cohort study with an extensive baseline examination and a periodic longitudinal follow-up. The current article provides a comprehensive synopsis for the main design of the SENIORLAB study, including a report on participant recruitment, baseline examinations, and follow-up.

### 2.2 Setting

The study was initiated by a medical laboratory and aimed to recruit study participants in the community. The location and organization of the study center is associated with the laboratory, which is located in Berne in the midlands of Switzerland. For the baseline examination, study participants were recruited from May 2009 to December 2011. Follow-up examinations are planned every 3 to 5 years. The first follow-up was done from December 2013 to December 2014. The second follow-up investigation is scheduled for October 2017 to October 2018. The study is registered in the International Standard Randomized Controlled Trial Number registry (ISRCTN53778569), where all items of the World Health Organization (WHO) Trial Registration Data Set have been specified.

### 2.3 Ethics, consent, and permissions

The study was approved by the Ethics Committee Bern (Kantonale Ethikkommission Bern; ref 166/08) on January 5, 2009, and an amendment regarding the prospective study was approved on July 29, 2013. Study participants provided written informed consent before entering the study.

### 2.4 Participants

Consecutive, subjectively healthy elderly volunteers aged 60 years and older were recruited. The study participants were contacted through newspaper advertisements, various clubs, and at associations that had high proportions of healthy elderly members (e.g., alpine clubs and sports clubs). In addition, participants were recruited through personal contacts of the collaborators of the study organization. Inclusion criteria were as follows: age 60 or older, residence in Switzerland, the subjective perception of being healthy, and being in a fasting state at the baseline examination. Exclusion criteria were as follows: known diabetes mellitus, known thyroid disease, current glucocorticoid use, active neoplastic disease during the past 5 years, consumption of >5 pharmacologically active substances (polypharmacy), and hospitalization during the past 4 weeks. Study participants and their primary care physicians, as requested, were given the results of selected values that are widely screened in asymptomatic healthy individuals, such as fasting glucose, total cholesterol, creatinine, hemoglobin, red blood cells, white blood cells, and thrombocytes. For the follow-up examinations, study participants are contacted by letter and telephone. In the case of a participant's lack of response, official communal authorities, relatives, and/or neighbors are contacted to obtain the study participant's information.

### 2.5 Data collection and measurements

Each participant's personal history and anthropometric measurements, such as height and weight, were obtained. Histories of participants were obtained using questions including whether a participant considered himself or herself as healthy and believed to have an intact cognitive state. Furthermore, a personal history of diseases and surgical procedures, as well as intake of drugs and supplements, was recorded. Blood pressure was measured in a standardized manner in a seated position after a 10-minute rest. Venous blood was drawn into S-Monovette (Sarstedt, Sevelen, Switzerland) after an overnight fasting period. The laboratory samples were processed (i.e., centrifuged, aliquoted, and analyzed or frozen at −80°C) immediately after the blood was drawn to allow for standardized preanalytics. In the follow-up surveys, information on subjective well-being, mortality, reason of death, hospitalization, and impaired autonomy is recorded.

The investigators will have access to the final dataset. For qualified analysis plans, they will provide data access to qualified scientists. The data monitoring committee consists of the 3 study investigators and ascertains integrity as well as correctness of data and analyzed and handled adverse events and other unintended effects. This was done after the baseline examination and will be continued after each follow-up period. Follow-up will be performed until funds are available. Confidentiality of data is protected before, during, and after the trial.

Laboratory parameters were measured on up-to-date diagnostic platforms from different manufacturers (e.g., Roche Diagnostics, Abbott Diagnostics, Siemens, Beckmann Coulter, Sysmex) and also by employing, for example, open immunoassay platforms or high-performance liquid chromatography-tandem mass spectrometry instruments. The laboratory analyses were performed according to the requirements of the Swiss commission for quality assurance in the medical laboratory. For internal quality control, commercially available control materials were used when available. The investigated parameters, together with manufacturer and imprecision, are provided in Table 1  . If available, aliquots of each sample were stored at −80°C. This step allows for the analysis of additional parameters of interest. The freezers used to store the samples have been continuously monitored for an adequate temperature.

**Table 1:**
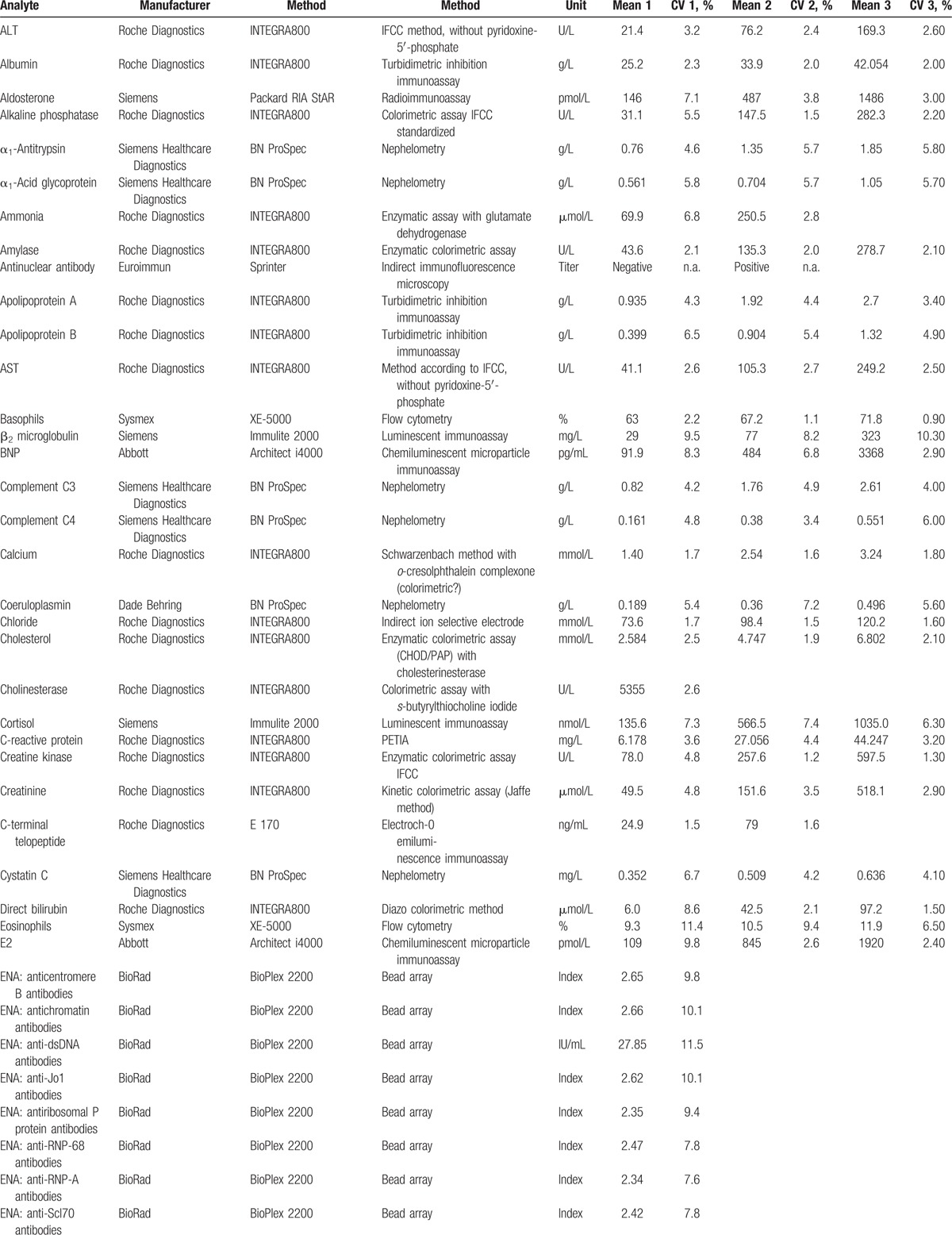
Employed laboratory methods with their imprecision at different concentration levels.

**Table 1 (Continued):**
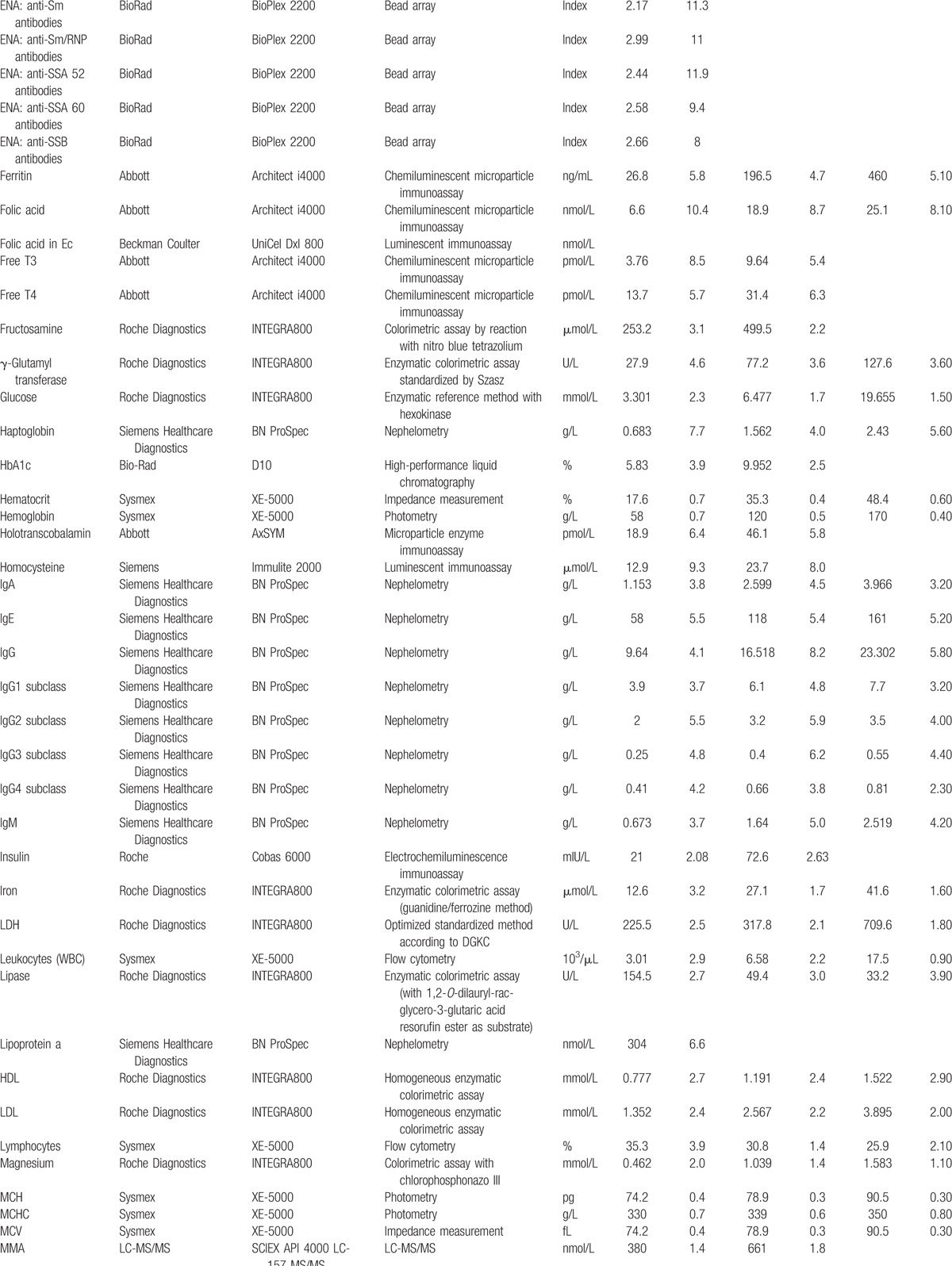
Employed laboratory methods with their imprecision at different concentration levels.

**Table 1 (Continued):**
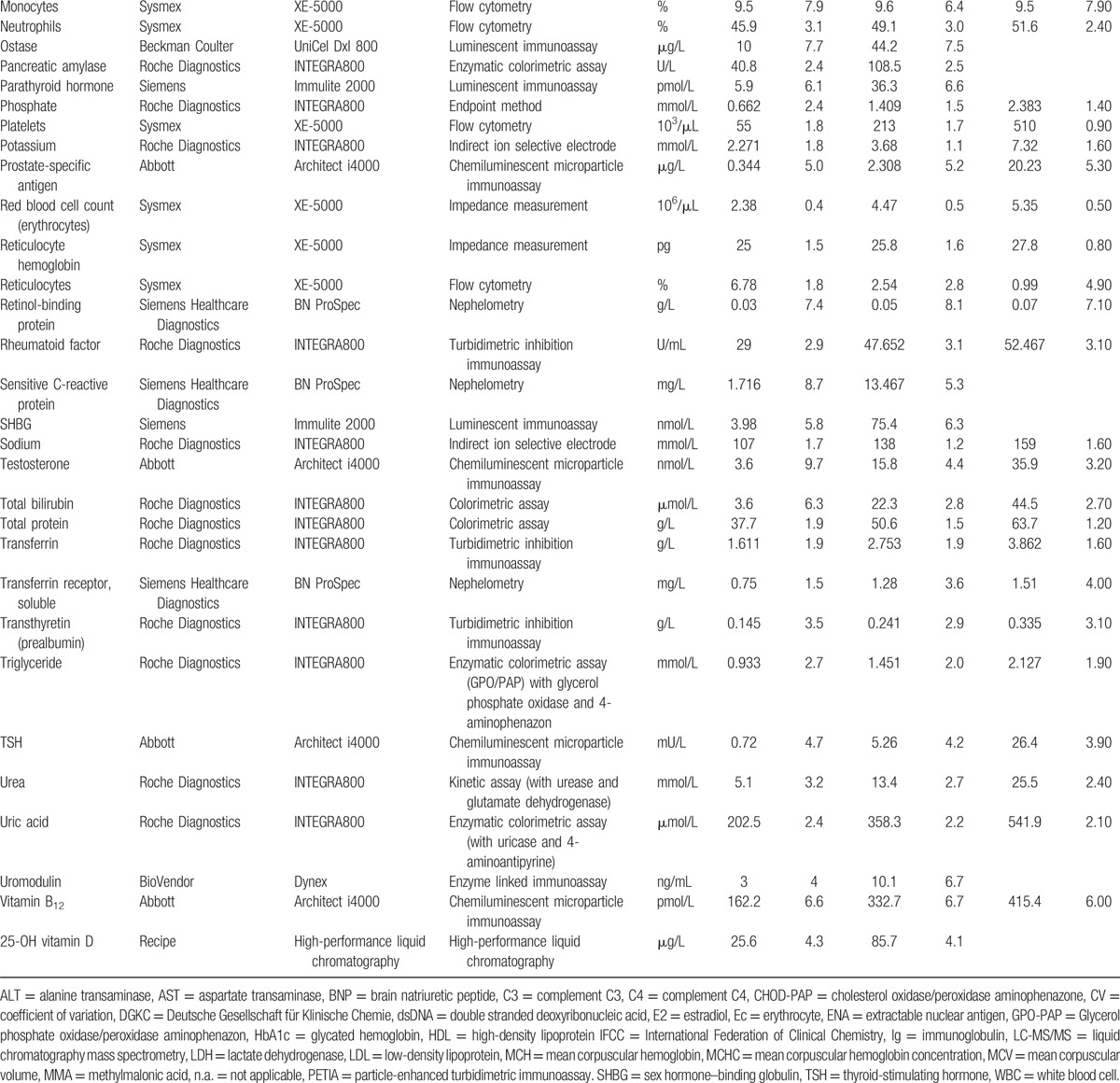
Employed laboratory methods with their imprecision at different concentration levels.

### 2.6 Sample size

The Clinical Laboratory Standards Institute (CLSI) released a relevant guideline (C28-A3c) for the evaluation of reference intervals.^[15]^ There are several possibilities to evaluate reference intervals. The most commonly stated minimal number of included subjects is 120 per stratum. This number allows for determination of the 90% confidence interval of the upper and lower reference limits. However, the so-called robust method also offers the possibility to evaluate reference intervals with fewer individuals. We anticipated age and gender stratification. For age stratification, we collapsed the participants to 5-year age cohorts (e.g., age 60–64, 65–69, 70–74). We aimed to include at least 120 participants in the 5-year age cohorts. The recruitment for an age stratum was stopped if >200 participants were already included in the respective stratum.

### 2.7 Analysis plan

**Figure 1:**
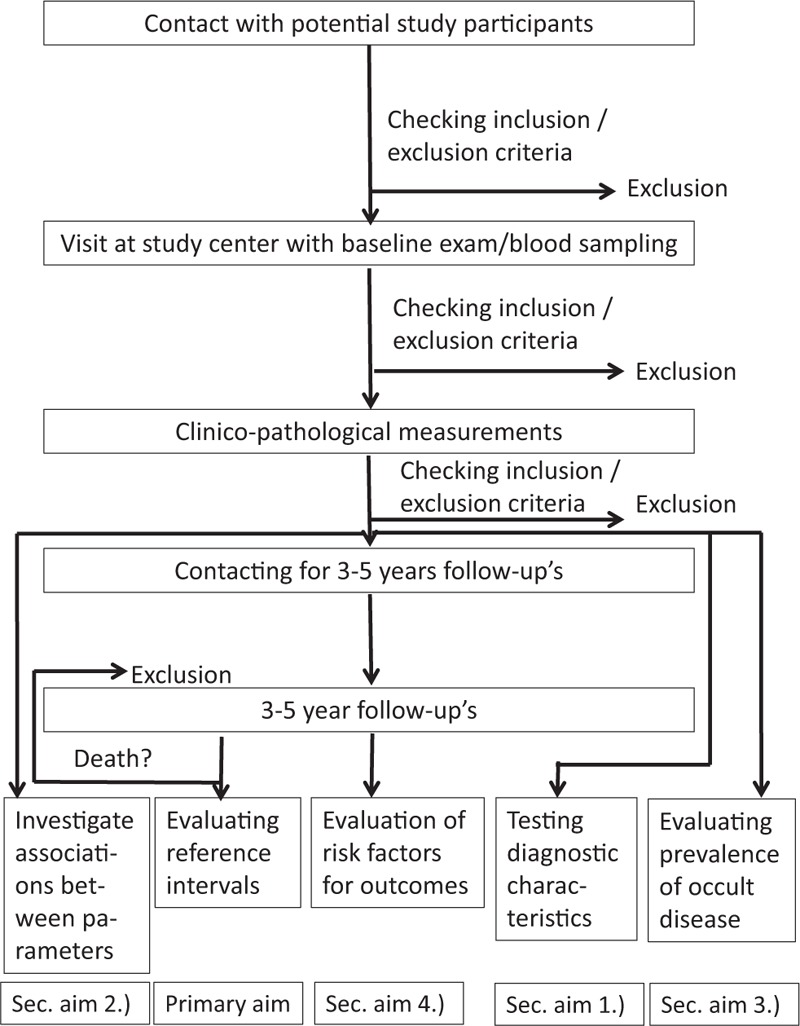
Study algorithm of the SENIORLAB study.

An overview on participant enrollment, data acquisition, and data analysis is given in the flow diagram (see Fig. 1). Participants can be excluded at different stages of the study: before baseline examination or after the baseline visit. Clinicopathological measurements were conducted immediately after drawing the blood samples to provide optimal preanalytical conditions. Due to logistical reasons, it sometimes only became apparent that an exclusion criterion applied after clinicopathological measurements were conducted (e.g., in the case of consumption of a combination drug or in the case of the consumption of an antidiabetic medication in the absence of information that a participant suffers from diabetes, despite explicitly being asked about this condition). Together, 1467 individuals presented at the baseline visit. For the determination of reference intervals, participants lost to follow-up will not be included into the statistical analysis.

To evaluate reference intervals (primary aim) according to the CLSI C28-A3c guideline, laboratory parameters will be analyzed for partitioning factors (e.g., age, gender) by correlation in the case of continuous variables and by a statistical comparison of the means and medians in the case of binary variables. Outliers will be eliminated according to the method of Dixon and Reed or alternative methods.^[15]^ After an analysis of the normal distributions of the laboratory results, parametric or nonparametric reference ranges will be calculated. Should a stratum have fewer than 120 subjects, then the robust method will be applied. Judgment on whether the included number of study participants in a respective stratum is sufficient will be made by the method of Boyd and coworkers.^[15]^ Furthermore, age-related continuous reference intervals are calculated.^[28–30]^ The computer programs MedCalc (Mariakerke, Belgium) and StatisPro (CLSI, Wayne, PA) will be used for these calculations and analyses.^[15]^

For statistical analysis of the secondary aims, the following methods will be used: diagnostic characteristics will be evaluated by Receiver operating characteristic curve analysis, and sensitivity, specificity, predictive values, and likelihood ratios will be calculated (by looking at optimum decision concentrations and concentrations with high sensitivity and high specificity, this approach may be able to evaluate decision limits that identify persons with probable or improbable disease and describe a gray zone of diagnostic uncertainty)^[31]^; associations between different variables will be assessed by correlation and by univariate and multivariate linear regression analyses (the latter will represent a means to control for covariates, confounders, or potential bias; goodness of model fit, regression coefficients, and partial correlation coefficients will be evaluated in these models); descriptive statistics will be used to evaluate the frequency of occult disease; logistic regression analysis and longitudinal analysis will be employed to evaluate risks for the occurrence of outcomes.

For all other analyses, statistical comparisons will be performed by *t* test/Mann–Whitney *U* test, χ^2^ or Fisher exact test; tests for trend; or analysis of variance/Kruskal–Wallis test, as indicated by the specific statistical problem. The computer programs MedCalc (Mariakerke) and SPSS (IBM, Zurich, Switzerland) will be used for the analyses of secondary aims. Study results will be disseminated via publications. Authorship will be attributed according to the criteria issued by the International Committee of Medical Journal Editors. Data will be made accessible to other researches after data sharing arrangements could be met.

## 3 Discussion

To our knowledge, the SENIORLAB study represents the largest and most comprehensive study aiming at establishing reference intervals in the elderly thus far. Typically, reference interval studies are cross-sectional in nature and are conducted in healthy cohorts. According to the WHO, health is a status of subjectively perceived well-being: “health is a state of complete physical, mental and social well-being and not merely the absence of disease or infirmity.”^[32,33]^ Because seniors frequently suffer from ≥1 diseases without any effect on the subjective perception of health status (e.g., arterial hypertension), the definition of health represents a crucial and problematic point in establishing reference intervals in this age group.^[34]^ Normal senior reference subjects without any disease, medication, and supplementation are too rare and cannot be regarded representative for the group of “healthy seniors.”^[35]^ Therefore, novel approaches for selecting reference subjects are warranted for establishing reference intervals in seniors. In addition to the subjective perception of health, the SENIORLAB study, with its longitudinal design, adds objectifying characteristics such as survival and long-term well-being.

The secondary aims of the SENIORLAB study will be able to clarify the physiology of different parameters, as it has already been illustrated by describing the importance of serum uromodulin, an analyte that is increasingly recognized for its importance in understanding the development of renal and cardiovascular disease and kidney function in healthy persons.^[36]^ Furthermore, parameters can be identified for the screening of commonly encountered disorders among the elderly population, such as using hematological indices to screen for common nutritional deficiencies of different vitamins and iron. Such an approach may help to increase the effective use of resources in the clinical laboratory. Associations between different parameters will help to better understand the pathophysiology of different parameters and allow for better interpretation.^[37,38]^ Finally, the investigation of the prevalence of occult disease in the elderly and the identification of longitudinal risks, such as mortality, is of great importance from a public health perspective. This approach is likely to indicate possibilities to antagonize adverse outcomes for individuals and the public. Together, the SENIORLAB study can provide important evidence in the following ways: for the principle and practice of using laboratory medicine resources, for identification of better index and screening tests, for better understanding of physiological behavior of laboratory parameters, for identifying underdiagnosed and undervalued diseases in the elderly, and for identifying important risk factors in the elderly.

The SENIORLAB study possesses strengths and limitations. Regarding the primary aim of reference interval determination, internal validity is hampered by the fact that biological variation was not considered because, in most individuals, only 1 blood sample was obtained per participant and only a minority of participants provided 2 blood samples.^[39]^ As a consequence, the study cannot assess a preference of population-based reference intervals over intraindividual reference changing values.^[7]^ External validity is impaired by the fact that the investigated cohort consists entirely of participants with Caucasian descent. As a consequence, the evaluated reference intervals might not be extrapolated to persons with other racial backgrounds. Furthermore, the term of a subjectively healthy well-being is debatable and might also impair extrapolation to other groups. We intentionally chose a nonrestrictive definition of health, which in our view is closer to the majority of elderly persons. Absence of disease or medication is the elderly is relatively rare. For example, normal blood pressure is encountered in only 7% of persons aged ≥80 years.^[40]^ Furthermore, medication use in the elderly occurs very frequently, and surveys have shown that up to 80% in the age group of >65 years reported having ingested at least 1 drug during the past week.^[41]^

The fact that a longitudinal follow-up has been undertaken differentiates the SENIORLAB study from all other studies investigating reference intervals in the elderly. In this age group, a life expectancy >3 years can be considered as a long-term life expectancy.^[42,43]^ Based on the difficulties in performing a clear-cut objective differentiation of health from disease in the elderly, we are convinced that long-term life expectancy in addition to perception of subjective health is a robust and suitable additive criterion to select individuals for the evaluation of reference intervals. This approach is well supported by the findings from the Cardiovascular Health Study, which demonstrated that elderly study participants >65 years who were in the lowest hemoglobin quintile had an increased risk for death in a fully adjusted model despite not fulfilling WHO but also other criteria for anemia (i.e., hemoglobin concentration below the lower limit of the hemoglobin reference interval).^[44–46]^ This study clearly suggests that mortality should be considered when defining reference ranges in the elderly.

We expect the SENIORLAB study to provide insights on the influence of age on the behavior of laboratory parameters. Accordingly, we will be able to define accurate reference intervals and decision limits for a multitude of laboratory parameters, which constitute a great majority of clinically ordered laboratory test in senior citizens. From a methodological perspective, we will be able to include longitudinal follow-up as a relevant criterion to select appropriate individuals for the evaluation of reference intervals. This follow-up will be an important step in the theory and practice of defining reference intervals in the elderly.
